# Supplementary figures and images for: Salmonella Typhimurium impairs glycolysis-mediated acidification of phagosomes to evade macrophage defense
Source: PLoS Pathog. 2021 Sep 23;17(9):e1009943. doi: 10.1371/journal.ppat.1009943 (PMC8491875; doi:10.1371/journal.ppat.1009943)

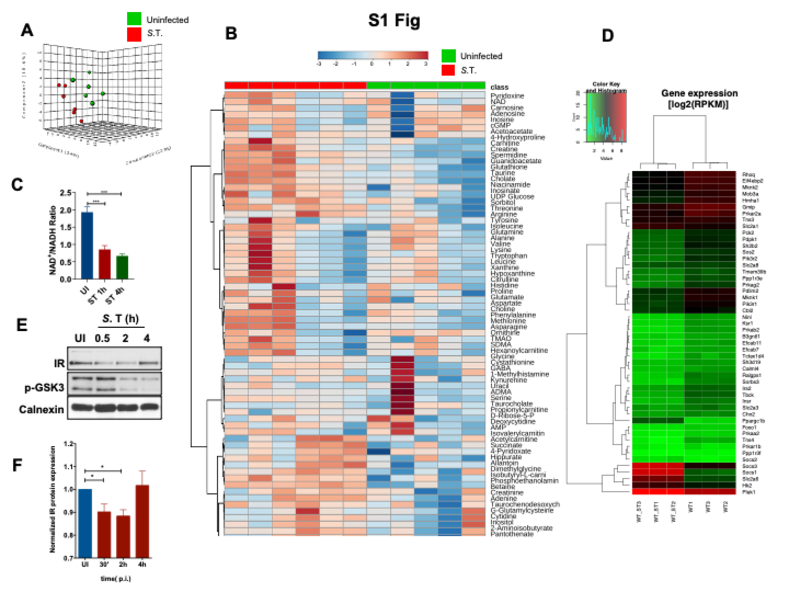

Supplement: S1 Fig — (A) Partial least squares discriminate analysis (PLS-DA) score plot representing the differential metabolomics profiles upon S. Typhimurium infection (S.T., 2h p.i.) compared to uninfected controls (n = 6). (B) Heatmap representation of 2-way hierarchical clustering of all altered metabolites analyzed from BMDMs upon S. Typhimurium infection (2h p.i.) compared to uninfected controls (n = 6). (C) Relative quantification of NAD+/NADH ratio of uninfected (UI) and S. Typhimurium-infected BMDMs (S.T., 1h and 4h p.i.) by targeted MS-UPLC analysis (n = 6). (D) Hierarchical clustering of the major differentially regulated transcripts in BMDMs infected with S. Typhimurium (2h p.i) compared to uninfected controls (n = 3). (E) Western blot analysis of insulin receptor and its downstream effector phospho-GSK3β; calnexin was used as a loading control. (F) Expression of insulin receptor was quantified from immunoblots using ImageJ and its relative expression to loading control is shown (n = 4). Data are shown as mean ± S.E.M. and statistical significance calculated using student t-test is represented as * = p<0.05; ** = p<0.01; *** = p<0.001. (TIF) [file ppat.1009943.s001.tif]

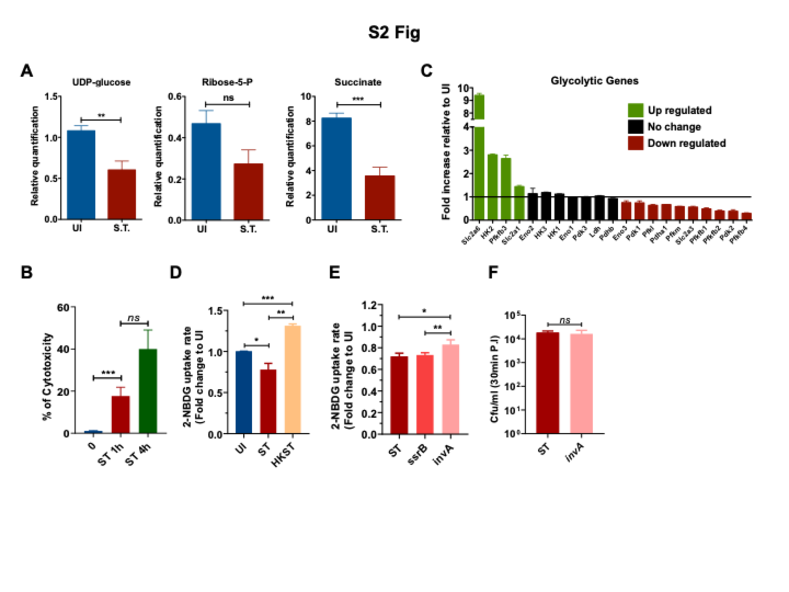

Supplement: S2 Fig — (A) Relative quantification of UDP-glucose, ribose-5-phosphate and succinate levels in S. Typhimurium-infected BMDMs (2h p.i.) by targeted MS-UPLC analysis (n = 6). (B) Cytotoxicity of S. Typhimurium towards BMDMs was determined by measuring lactate dehydrogenase (LDH) in the cell supernatant at 1 and 4h post-infection. (C) Expression levels of the glycolytic genes detected in the RNA-seq analysis represented a fold increase upon S. Typhimurium infection relative to uninfected controls (UI). (D) Glucose intake in S. Typhimurium-infected BMDMs (S.T.) and heat-killed S. Typhimurium-treated BMDMs (HK S.T.) (E) WT, ssrB and invA mutant S. Typhimurium for 2h represented as the fold increase in 2-NBDG MFI relative to uninfected (UI) controls analyzed by flow cytometry (n = 6). Data are shown as mean ± S.E.M. and statistical significance calculated using student t-test is represented as * = p<0.05; ** = p<0.01; *** = p<0.001. (F) Bacterial intake of WT and invA mutant S. Typhimurium in BMDMs measured by colony counting assay. The time point Zero, i.e immediately after washing off extracellular bacteria was used. (TIF) [file ppat.1009943.s002.tif]

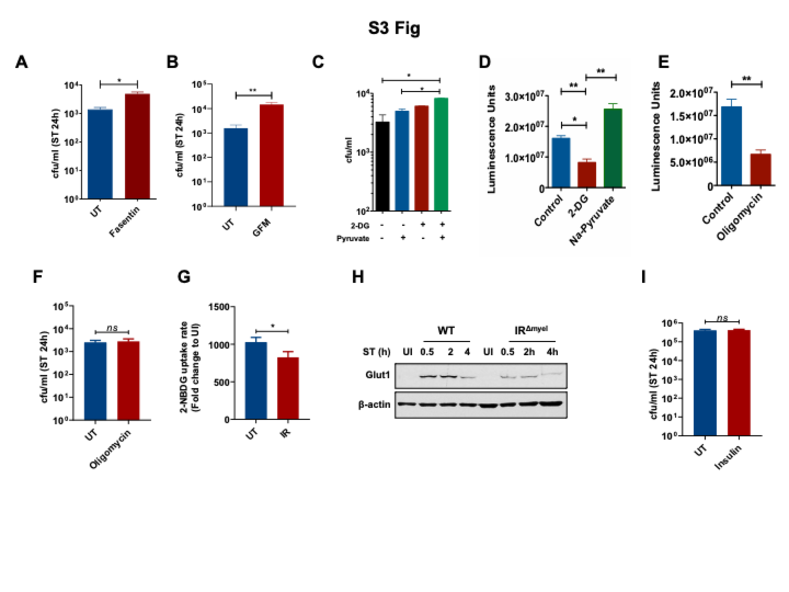

Supplement: S3 Fig — (A) Quantification of intracellular S. Typhimurium burden at 24h post-infection in untreated (UT) and fasentin-treated BMDMs (n = 3) (B) BMDMs grown in Glucose Free Medium (GFM) (n = 3). (C) S. Typhimurium burden in BMDMs pre-treated with 2-DG or Sodium pyruvate or both were represented as colony forming units (CFU) 24h post-infection. (D) Luminiusence of 2-DG and sodium pyruvate (E) oligomycin were analyzed using Promega CellTiter-Glo luminescent assay (F) S. Typhimurium burden in BMDMs from oligomycin treated BMDMs analyzed 24h post-infection. (G) Glucose uptake in WT and IRΔmyel BMDMs measured as 2-NBDG mean fluorescence intensity (MFI) by flow cytometry (n = 3). (H) Western blot analysis of Glut1 expression from WT and IR KO BMDMs at the indicated time points. The image shown is representative of 3 individual experiments (I) Intracellular S. Typhimurium levels in UT and recombinant insulin pre-treated BMDMs 24h post-infection. (TIF) [file ppat.1009943.s003.tif]

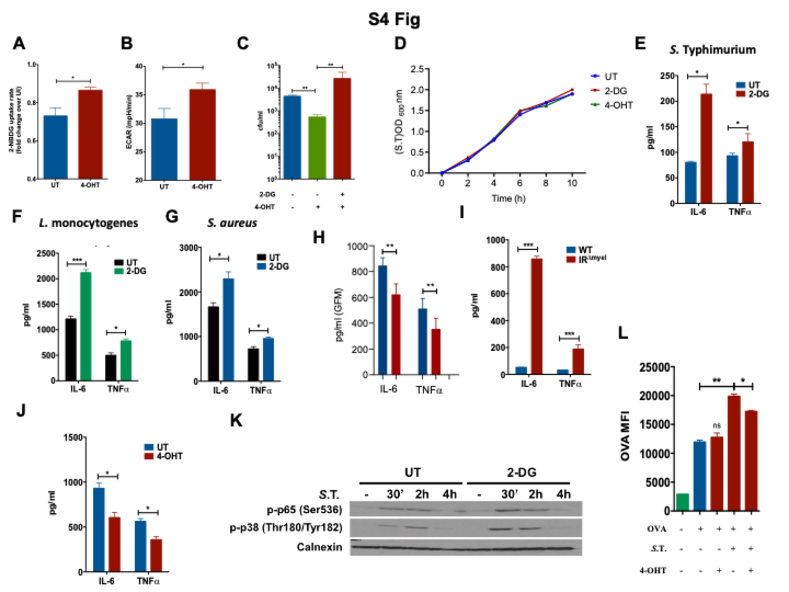

Supplement: S4 Fig — (A) Glucose uptake in S. Typhimurium-infected and 4-OHT-treated BMDMs compared to infected and untreated controls (UT) measured as 2-NBDG MFI by flow cytometry (n = 3). 2-NBDG was acquired at 2h post-infection. Data is normalized to uninfected controls. (B) Sea Horse measurement of ECAR in 4-OHT-treated BMDMs compared to untreated controls (n = 6). (C) Quantification of intracellular S. Typhimurium burden at 24h post-infection in untreated (UT) BMDMs compared to BMDMs simultaneously treated with 2-DG and 4-OHT (n = 3). (D) Growth curves of S. Typhimurium measure at ID600 in the presence of 2-DG or 4-OHT in LB medium supplemented with streptomycin. (E) IL-6 and TNF-α secretion by 2-DG-treated macrophages infected with S. Typhimurium (F), L. monocytogenes (G) or S. aureus represented 24h post-infection. (H) IL-6 and TNF-α levels measured from macrophages infected with S. Typhimurium in Glucose Free Medium (I) IL-6 and TNFα in supernatants of IRΔmyel BMDMs and WT infected with S. Typhimurium for 24h (n = 3). (J) IL-6 and TNFα in supernatants from 4-OHT-treated BMDMs after 24h of infection with S. Typhimurium (n = 3). (K) Western blot analysis of phospho-p65 and phospho-p38 in 2-DG-pre-treated and S. Typhimurium-infected BMDMs compared to untreated (UT) controls. The image shown is representative of 3 individual experiments. (L) MFI of unprocessed Alexa647-labelled OVA in bead containing phagosomes from 4-OHT-treated and S. Typhimurium-infected BMDMs analyzed by flow cytometry (n = 3). Data are shown as mean ± S.E.M. and statistical significance calculated using student t-test is represented as * = p<0.05; ** = p<0.01; *** = p<0.001. (TIF) [file ppat.1009943.s004.tif]

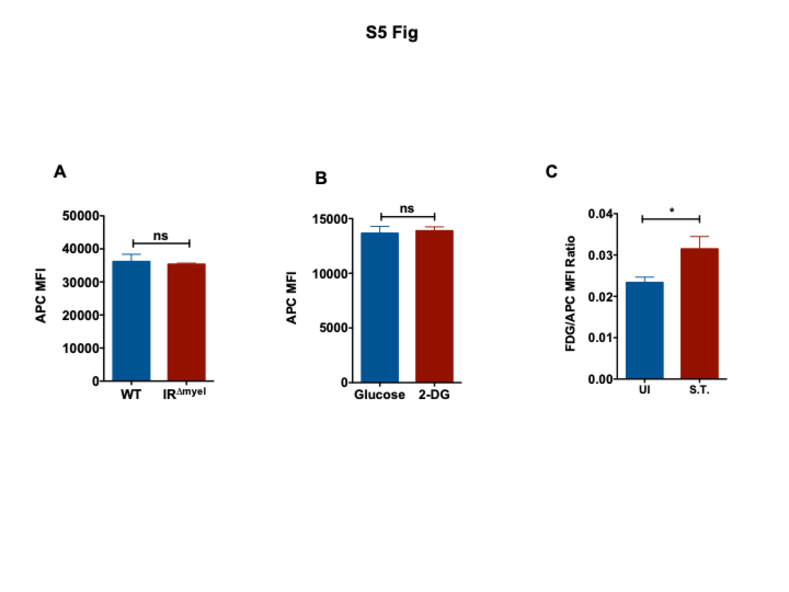

Supplement: S5 Fig — (A) Intake of fluorescent beads (APC) by WT and IRΔmyel BMDMs shown as mean fluorescent intensity (MFI) analyzed by flow cytometry (n = 3). (B) Intake of fluorescent beads in 2-DG-treated BMDMs compared to untreated BMDMs analyzed by flow cytometry and shown as MFI (n = 3). (C) Flow cytometry analysis of C12FDG and Alexa647-coated beads in S. Typhimurium-infected BMDMs for 30min compared to uninfected (UI) BMDMs (MFI of C12FDG normalized to Alexa647 MFI) (n = 3). Data are shown as mean ± S.E.M. and statistical significance calculated using student t-test is represented as * = p<0.05; ** = p<0.01; *** = p<0.001. (TIF) [file ppat.1009943.s005.tif]

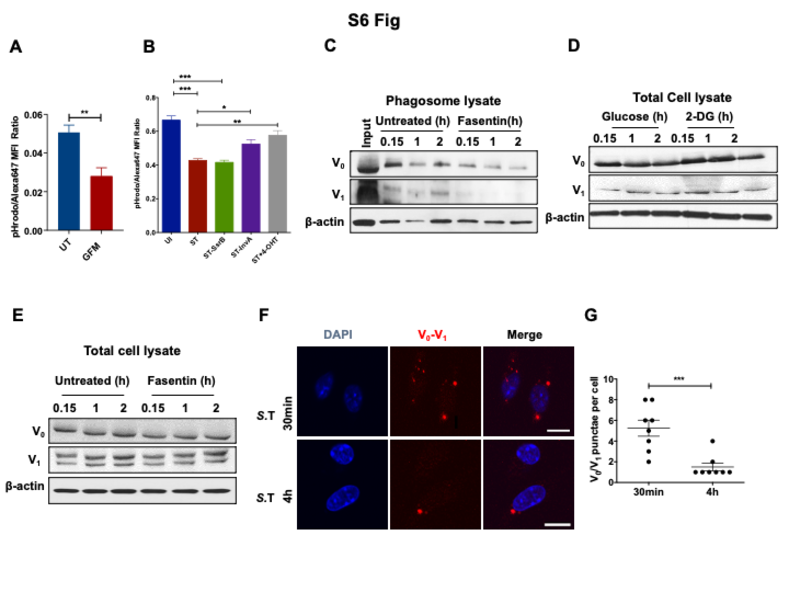

Supplement: S6 Fig — (A) MFI of pHrodo-E. coli particles in S. Typhimurium infected WT BMDMs grown in Glucose-free medium (B) MFI of pHrodo-E. coli particles in WT, ΔinvA, ΔssrB mutant S. Typhimurium infected BMDMs and 4-OHT treated BMDMs infected with S. Typhimurium (2h p.i.) normalized to Alexa647 MFI (n = 3). (C) Western blot analysis of v-ATPase subunits V0 and V1 in isolated phagosomes from fasentin-treated BMDMs. The image shown is representative of 3 individual experiments. (D) Western blot analysis of total levels of the v-ATPase subunits V0 and V1 in 2-DG-treated-BMDMs. The image shown is representative of 3 individual experiments. (E) Western blot analysis of total levels of the v-ATPase subunits V0 and V1 in fasentin-treated-BMDMs. (F) PLA analysis of v-ATPase subunits V0 and V1 interaction in 2-DG-treated BMDMs infected with S. Typhimurium using confocal microscopy (scale bars indicate 10 μm). The image shown is representative of 3 individual experiments. (G) Quantification of V0 and V1 interaction in 2-DG treated BMDMs (n = 15). Data are shown as mean ± S.E.M. and statistical significance calculated using student t-test is represented as * = p<0.05; ** = p<0.01; *** = p<0.001. (TIF) [file ppat.1009943.s006.tif]

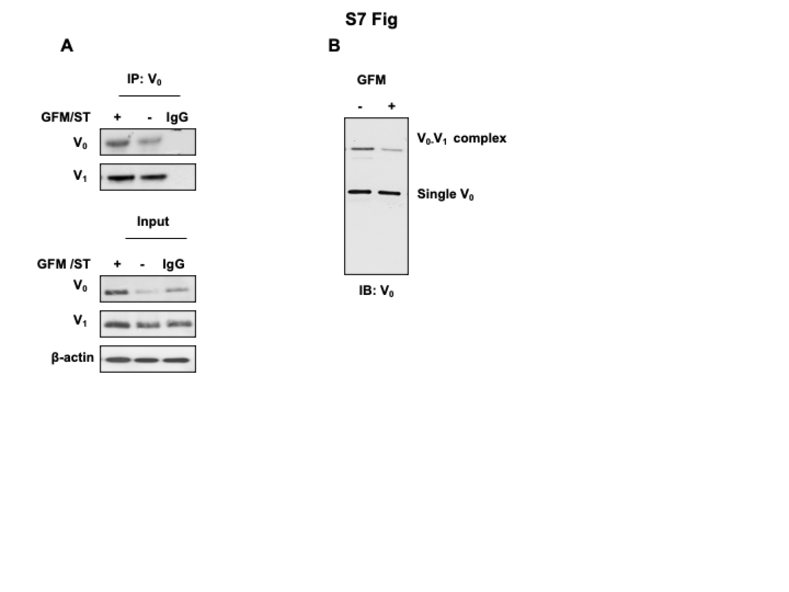

Supplement: S7 Fig — (A) Immunoprecipitation of V0 subunit from isolated bead phagosomes grown in glucose-free medium analyzed for V0 and V1 subunits. (B) Native PAGE analysis of isolated bead phagosome from BMDMs grown in Glucose free medium (GFM). (TIF) [file ppat.1009943.s007.tif]

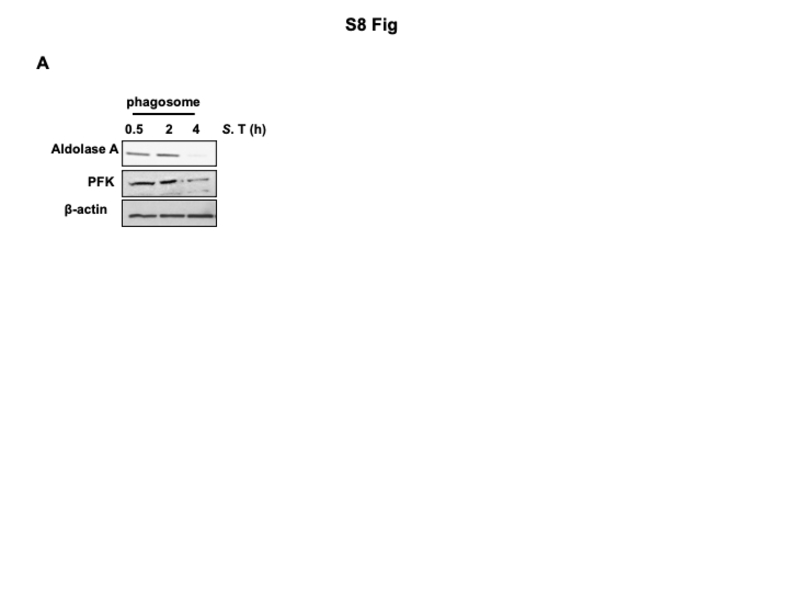

Supplement: S8 Fig — (A) Immunoblot from isolated S. Typhimurium phagosomes probed for Aldolase A and phosphofructokinase (PFK) and actin at indicated time points. (TIF) [file ppat.1009943.s008.tif]
