# Supplementary material for: Salmonella Typhimurium impairs glycolysis-mediated acidification of phagosomes to evade macrophage defense
Source: PLoS Pathog. 2021 Sep 23;17(9):e1009943. doi: 10.1371/journal.ppat.1009943 (PMC8491875; doi:10.1371/journal.ppat.1009943)
Supplement: S1 Table — (PDF) [file ppat.1009943.s009.pdf]

S1 Table

Isotopically labeled internal standards were obtained from Cambridge Isotope Laboratory. Inc., USA (Ordered from Euroiso-Top, France). We have used total of 12 internal standards to compensate loss of metabolites either during extraction or analysis in mass spectrometry. List of internal standards with their concentration were given below:

IST compound	Concentration of Intermediate (ppm)
L-Glutamic acid U-13C5	487,042
L-Alanine D7	2075,637
L-Serine D3	118,775
L-ArginineHCL U-13C6	370,232
L-Lysine:2HCL U-13C6	372,591
L-Proline U-13C5, 15N	454,225
L-Phenylalanine U-13C9, 15N	787,977
L-Methionine U-13C5, 15N	226,828
L-Isoleucine D10, 15N	256,048
Guanosine U-15N5	41,483
Panthotenic acid, Ca salt Monohydrate 13C3, 15N	108,474
Cytosine U-13C2, 15N3	31,055

The mixed working solution for IST is prepared by taking equal volumes of each of the 12 individual IST intermediate solutions and mixing with vortex. For example, 100 µl of each IST intermediate solution is taken and mixed to get a total of 1.2 ml of IST working solution. The working solution is stored at $+4^{0}$C.
